# SIRT1 Promotes Cisplatin Resistance in Bladder Cancer via Beclin1 Deacetylation-Mediated Autophagy

**DOI:** 10.3390/cancers16010125

**Published:** 2023-12-26

**Authors:** Yan Sun, Xudong Liu, Hang Tong, Hubin Yin, Tinghao Li, Junlong Zhu, Junrui Chen, Linfeng Wu, Xiaoyu Zhang, Xin Gou, Weiyang He

**Affiliations:** Department of Urology, The First Affiliated Hospital of Chongqing Medical University, Chongqing 400016, China; 2021130038@stu.cqmu.edu.cn (Y.S.); 2020440038@stu.cqmu.edu.cn (X.L.); 2019010062@cqmu.edu.cn (H.T.); 204625@hospital.cqmu.edu.cn (H.Y.); 2022140056@stu.cqmu.edu.cn (T.L.); 2023140143@stu.cqmu.edu.cn (J.Z.); 2021110203@stu.cqmu.edu.cn (J.C.); 2022110209@stu.cqmu.edu.cn (L.W.); 2022110221@stu.cqmu.edu.cn (X.Z.); gouxincq@hospital.cqmu.edu.cn (X.G.)

**Keywords:** bladder cancer, SIRT1, autophagy, deacetylation, cisplatin resistance

## Abstract

**Simple Summary:**

This investigation delves into the intricate mechanisms of autophagy-dependent cisplatin resistance in bladder cancer. Our primary objective is to unveil the precise role of SIRT1, a deacetylase enzyme, in orchestrating cisplatin resistance through autophagic pathways in the cisplatin-resistant T24/DDP cell line. The findings demonstrate that inhibiting autophagy diminishes drug resistance, while SIRT1 overexpression intensifies this resistance by stimulating autophagy. Significantly, silencing SIRT1 elevates Beclin1 acetylation, suppressing autophagy and attenuating cisplatin resistance. This nuanced understanding of SIRT1-mediated cisplatin resistance not only furthers our theoretical foundation, but also furnishes potential therapeutic targets.

**Abstract:**

Autophagy-dependent cisplatin resistance poses a challenge in bladder cancer treatment. SIRT1, a protein deacetylase, is involved in autophagy regulation. However, the precise mechanism through which SIRT1 mediates cisplatin resistance in bladder cancer via autophagy remains unclear. In this study, we developed a cisplatin-resistant T24/DDP cell line to investigate this mechanism. The apoptosis rate and cell viability were assessed using flow cytometry and the CCK8 method. The expression levels of the relevant RNA and protein were determined using RT-qPCR and a Western blot analysis, respectively. Immunoprecipitation was utilized to validate the interaction between SIRT1 and Beclin1, as well as to determine the acetylation level of Beclin1. The findings indicated the successful construction of the T24/DDP cell line, which exhibited autophagy-dependent cisplatin resistance. Inhibiting autophagy significantly reduced the drug resistance index of these cells. The T24/DDP cell line showed a high SIRT1 expression level. The overexpression of SIRT1 activated autophagy, thereby further promoting cisplatin resistance in the T24/DDP cell line. Conversely, inhibiting autophagy counteracted the cisplatin-resistance-promoting effects of SIRT1. Silencing SIRT1 led to increased acetylation of Beclin1, the inhibition of autophagy, and a reduction in the cisplatin resistance of the T24/DDP cell line. Introducing a double mutation (lysine 430 and 437 to arginine, 2KR) in Beclin-1 inhibited acetylation and activated autophagy, effectively reversing the decreased cisplatin resistance resulting from SIRT1 silencing. In summary, our study elucidated that SIRT1 promotes cisplatin resistance in human bladder cancer T24 cells through Beclin1-deacetylation-mediated autophagy activation. These findings suggest a potential new strategy for reversing cisplatin resistance in bladder cancer.

## 1. Introduction

Bladder cancer is a prevalent malignant tumor of the urinary system in China, with increasing incidence and mortality rates in recent years [1]. Cisplatin-based chemotherapy remains a primary treatment approach for bladder cancer [2]. However, the development of cisplatin resistance poses a significant challenge, as this often hinders the achievement of optimal and long-lasting treatment outcomes [3]. Recent studies have highlighted the close association between the autophagy-mediated self-protection of tumor cells and cisplatin resistance [4]. Modulating the proteins involved in the autophagy pathway holds promise as a potential strategy for reversing cisplatin resistance.

Acetylation is an important post-translational modification, and many autophagy-related proteins have been shown to be regulated by acetylation [5]. SIRT1 is a nicotinamide adenine dinucleotide (NAD+)-dependent class III histone deacetylase involved in the regulation of the acetylation of several autophagy-related proteins, including LC3, ATG7, and ATG5 [6,7,8,9]. It plays a crucial role in autophagy regulation, chemotherapy resistance, drug development, and tumorigenesis [10,11]. However, the specific mechanism through which SIRT1 mediates cisplatin resistance in bladder cancer via autophagy remains unclear.

Beclin1 (BECN1) is the mammalian homolog of yeast Atg6 and serves as a pivotal protein in the early stages of autophagy initiation [12]. Research has indicated that cisplatin can induce protective autophagy by activating Beclin1 in human bladder cancer cells, highlighting the significance of Beclin1-dependent autophagy in cisplatin resistance in bladder cancer [13]. Furthermore, the deacetylation modification of Beclin1 is implicated in the activation of autophagy, with SIRT1 primarily mediating this deacetylation modification [6,14], opening up exciting avenues for therapeutic interventions aimed at manipulating autophagy for the treatment of various diseases.

Recent reports have suggested that the T24 cell line displays a relatively low responsiveness to cisplatin, making it a suitable model for investigating the mechanisms of cisplatin resistance in bladder cancer [15]. Therefore, this study selected the T24 cell line to establish the cisplatin-resistant T24/DDP cell line, aiming to deeply explore the potential mechanism through which the SIRT1–Beclin1 pathway regulates autophagy-dependent cisplatin resistance in bladder cancer. The findings of this study may offer a novel theoretical foundation and potential intervention target for the prevention and treatment of bladder cancer.

## 2. Materials and Methods

### 2.1. Construction and Culture of the T24/DDP Cell Lines

Human bladder cancer T24 cell lines were purchased from the Cell Bank of the Chinese Academy of Sciences, Shanghai. DMEM medium supplemented with 10% FBS was used, and the cells were grown in a 37 °C, 5% CO_2_ environment. The T24 cells were induced to be resistant to cisplatin by gradually increasing the cisplatin concentration in the medium to establish the T24/DDP cell line. The specific methods were as follows: the appropriate concentration of cisplatin was added to the medium of T24 cells in the logarithmic growth phase. The starting concentration of cisplatin was 0.1 μg/mL. After 48 h, cisplatin-containing media were discarded, fresh media were added, and the culture was continued. A higher concentration of cisplatin was added for 48 h after the cell proliferation rate was recovered. The above treatment was repeated in a culture of cells to make the T24 cells resistant to cisplatin through intermittent stepwise induction. Subsequently, the half maximal inhibitory concentrations (IC50) of cisplatin for the T24 and T24/DDP cell lines were determined using the CCK-8 kit, respectively. The resistance index (RI) was calculated based on the obtained results. RI = IC50_T24/DDP_/IC50_T24_.

### 2.2. RT-qPCR Assay

The primers used in the study were designed and synthesized by Tsingke (Beijing, China). The sequences are provided in Table 1. Human ACTB was used to serve as an internal control. The SIRT1 mRNA expression level was assessed using the quantification approach (2^−ΔΔCt^ method) relative to the ACTB expression levels.

### 2.3. Transfection of Plasmids and siRNAs

All targeted small interfering RNAs (siRNAs) were purchased from GenePharma (Shanghai, China). All over-expressed plasmids (empty plasmid vectors, pCDNA3.1-SIRT1, pCDNA3.1-Flag-Beclin1, and pCDNA3.1-Flag-Beclin1-2KR) were purchased from Tsingke (Beijing, China). Plasmids and siRNAs were transfected into the T24 or T24/DDP cell lines using Lipofectamine 2000 (Invitrogen, Carlsbad, CA, USA), according to the manufacturer’s instructions.

### 2.4. Lentivirus Transfection

To generate cell lines with silenced SIRT1 (shSIRT1) or controls (shNC), we employed a lentiviral system from Shanghai GenePharma Co., Ltd. (Shanghai, China). Lentivirus transduction was carried out when the T24 cells reached 50% confluence. The lentivirus, along with polybrene, was added to the cells at a multiplicity of infection of 50, following the manufacturer’s protocol. Polybrene was utilized to enhance the infection efficiency, and the transfection was conducted at 37 °C for 24 h. Post-transfection, the supernatant was replaced with RPMI-1640 and the cells were further cultured for 48 h at 37 °C. Subsequently, puromycin (2 μg/mL) was introduced into the medium to facilitate the stable selection of the transfected cell lines.

### 2.5. Cell Proliferation Assay

Cell proliferation was analyzed by performing Cell Counting Kit-8 (CCK-8) assays. Cells were seeded (5 × 10^3^ cells/plate) into 96-well plates and the concentration gradient for cisplatin intervention was set (0, 0.5, 1, 2, 4, and 8 μg/mL). After incubation for 48 h, the cells were incubated with CCK-8 reagent for 1 h in the dark. The number of viable cells was determined by measuring the absorbance at a wavelength of 450 nm with a microplate reader (Varioskan LUX; Thermo Fisher Scientific, Inc., Waltham, MA, USA).

### 2.6. Western Blot and Immunoprecipitation

The total protein was extracted using RIPA Lysis Buffer (Meilunbio, Dalian, China). Sodium dodecyl sulfate polyacrylamide gel electrophoresis (SDS-PAGE) was chosen for the total protein separation, and the proteins were then transferred to nitrocellulose membranes (the membranes were cut horizontally). The membranes were incubated with primary antibodies, including anti-p62/SQSTM1 (Abcam (Cambridge, UK), Cat# ab207305), anti-LC3B (Abcam, Cat# ab192890), anti-ACTB (Sangon Biotech (Shanghai, China), No. D110001), anti-PARP (Proteintech (Rosemont, IL, USA), Cat No. 13371-1-AP), anti-cleaved caspase3 (Cell Signaling Technology (Danvers, MA, USA), 5A1E), anti-SIRT1 (Cell Signaling Technology (Danvers, MA, USA), C14H4), anti-Acetylated lysine (Cell Signaling Technology (Danvers, MA, USA), #9441), and anti-Beclin1 (Proteintech, Cat No. 11306-1-AP). Immunoprecipitation was carried out either by incubating Anti-Flag beads (MCE (Addison, IL, USA), Cat. No. HY-K0207) at 4  °C with lysate overnight or by incubating an appropriate antibody with cell lysate for 4–6 h, followed by incubating Protein A/G immunoprecipitation beads overnight. Immunoprecipitates were washed three times with cold lysis buffer and eluted with SDS loading buffer by boiling for 10 min. The original western blot figures can be found in Figure S1.

### 2.7. Cell Immunofluorescence

Different groups of cells were fixed using 4% paraformaldehyde for 30 min, followed by three gentle washes with PBS. Subsequently, they were treated with 0.3% Triton X-100 for 15 min and washed three times gently with PBS. The cells were then incubated with an appropriate amount of 10% goat serum at room temperature for 30 min. Following the removal of the goat serum, diluted anti-LC3B (1:100) was added to the cells and incubated at 4 °C overnight. After three gentle washes with PBS, a diluted secondary antibody, Alexa 488-conjugated goat anti-rabbit IgG (Beyotime Cat# A0423), was added and incubated at room temperature for 1 h. After counterstaining with DAPI (Beyotime Cat# C1002), the cells were observed under a confocal microscope.

### 2.8. Flow Cytometry

The cells were harvested after intervention, washed twice with PBS, and then resuspended in 500 mL of PBS. The cells were transferred to 1.5 mL EP tubes, stained using the FITC Annexin V Apoptosis Detection kit II (BD Biosciences (San Jose, CA, USA), cat. No. 556570), and incubated in the dark for 10 min at room temperature, according to the manufacturer’s protocol. Subsequently, 10 µL of propidium iodide (PI, Beyotime Institute of Biotechnology) was added to the stained cells, and apoptotic cells were distinguished using a fluorescence-activated cell sorting analyzer (FACS; BD Biosciences). The results were analyzed with the Cytexpert V2.3 (Beckman Coulter, Inc., Brea, CA, USA) software. The apoptotic rate was calculated with the percentage of early + late apoptotic cells.

### 2.9. Mouse Xenograft Assays

The T24/DDP cells were transfected with lentivirus carrying either shSIRT1 to silence the SIRT1 gene or shNC as a control. The transfected T24/DDP cells (5 × 10^6^) were then injected into the flank of nude mice. The tumor dimensions were measured every 3 days using a caliper, and the tumor volume was calculated using the formula: length (L) × width (W)^2^ × 0.5, where L represents the longest diameter and W is the shortest diameter. After one week, an equivalent amount of DDP (2.5 mg/kg) or 0.9% normal saline was intraperitoneally injected every 3 days. The mice were euthanized at the end of the studies, and in vivo solid tumors were dissected and weighed. The animal study protocol was approved by the Institutional Animal Care and Use Committee (IACUC) of Chongqing Medical University (IACUC-CQMU-2023-0226).

### 2.10. Tissue Immunofluorescence and TUNEL Staining

Fresh tumor tissues were fixed in 4% paraformaldehyde and embedded in paraffin, then cut into 5 μm sections. This was followed by dewaxing, rehydration, antigen repair, and blocking, and the slides were incubated with the primary antibodies (SIRT1 Rabbit Recombinant mAb, Bimake, No. A5049) at 4 °C overnight. On day 2, secondary antibody Alexa 488-conjugated goat anti-rabbit IgG (Beyotime Cat# A0423) was added and incubated at room temperature for 1 h. After counterstaining with DAPI (Beyotime Cat# C1002), the cells were observed under a confocal microscope. TUNEL staining was performed using a One-step TUNEL In Situ Apoptosis Kit (Green, Elab Fluor^®^ 488, Elabscience, E-CK-A321) to detect apoptosis of the tumors, according to the manufacturer’s instructions. Images were captured via microscope and analyzed using Image J (v1.54f) [16].

### 2.11. Statistical Analysis

All experiments were independently repeated at least three times. The data were analyzed using the GraphPad Prism 8.0 software, and shown as the mean  ±  standard deviation (SD). A *t*-test was performed to compare the differences between two groups. A one-way analysis of variance was performed to analyze the differences among multiple groups. Statistical significance was set at *p*  <  0.05.

## 3. Results

### 3.1. Construction of the T24/DDP Cell Lines

**Figure 1:**
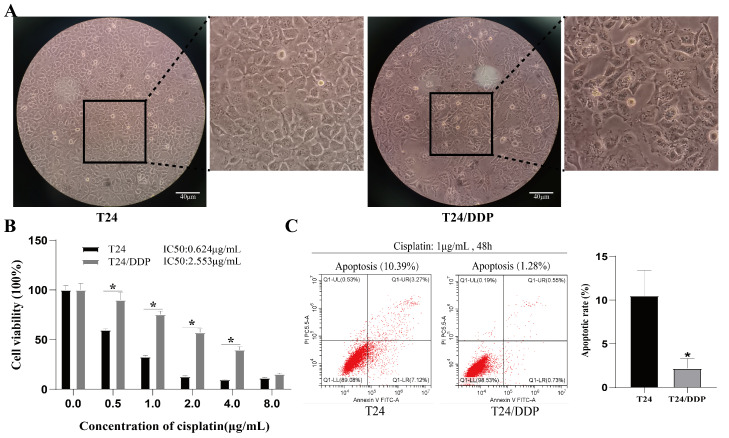
Construction of the T24/DDP cell lines. (**A**) Morphology of the T24 and T24/DDP cell lines under a microscope; scale bar: 40 µm. (**B**) IC50 of T24 and T24/DDP cell lines against cisplatin was detected with CCK 8 assay. (**C**) Apoptosis rate was determined with flow cytometry. * *p* < 0.05 signifies a statistically significant difference.

After 12 months, a cisplatin-resistant T24/DDP cell line was finally obtained, which grew and was passaged stably in culture medium containing 1 μg/mL of cisplatin. We observed that the T24 cells were round or oval, well-defined, of a uniform size, and displayed adherent growth. As the T24/DDP cells became larger and more irregular, the granules and vacuoles inside the cells became visible (Figure 1A). The CCK-8 results showed that the IC50 of the T24/DDP cell line was significantly higher than that of the T24 cell line, and the resistance index of the T24/DDP cell line to cisplatin was about 4.09 (Figure 1B). The T24 and T24/DDP cells were co-treated with 1 μg/mL of cisplatin for 48 h, resulting in a significantly elevated level of apoptosis in the T24 cells compared to the T24/DDP cells (Figure 1C).

### 3.2. Autophagy Mediates Cisplatin Resistance in Bladder Cancer

**Figure 2:**
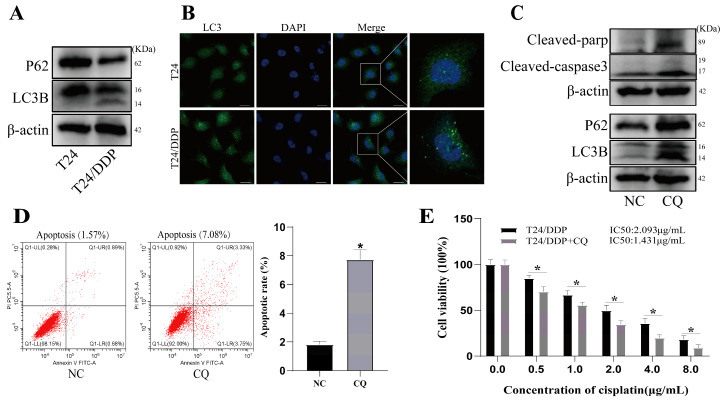
Autophagy mediates cisplatin resistance in bladder cancer. (**A**) Expression of autophagy marker proteins P62 and LC3B were determined with Western blot. (**B**) Autophagosome formation was detected with immunofluorescence; scale bar: 20 µm. (**C**) Expression of autophagy and apoptosis marker proteins were determined with Western blot. (**D**) Apoptosis rate was determined with flow cytometry. (**E**) IC50 of T24/DDP cell line against cisplatin was detected with CCK 8 assay. * *p* < 0.05 signifies a statistically significant difference.

Autophagy is an important mechanism of cisplatin resistance [2]. We measured the expressions of autophagy marker proteins in the T24 and T24/DDP cell lines using Western blot and immunofluorescence, and found increased autophagosome formation (Figure 2B), decreased P62 expression, and increased LC3 expression in the T24/DDP cell lines compared to the T24 cell lines (Figure 2A). After blocking autophagic flux with 20 μmol/L of CQ, the T24/DDP cells were found to be less able to maintain normal growth in medium containing 1 μg/mL of cisplatin; displayed significantly increased levels of both apoptosis (Figure 2D) and expressions of the apoptosis marker proteins Cleaved-parp and Cleaved-caspase3 (Figure 2C); and exhibited a decrease in the cisplatin resistance index (Figure 2E).

### 3.3. SIRT1 Mediates Cisplatin Resistance in Bladder Cancer

**Figure 3:**
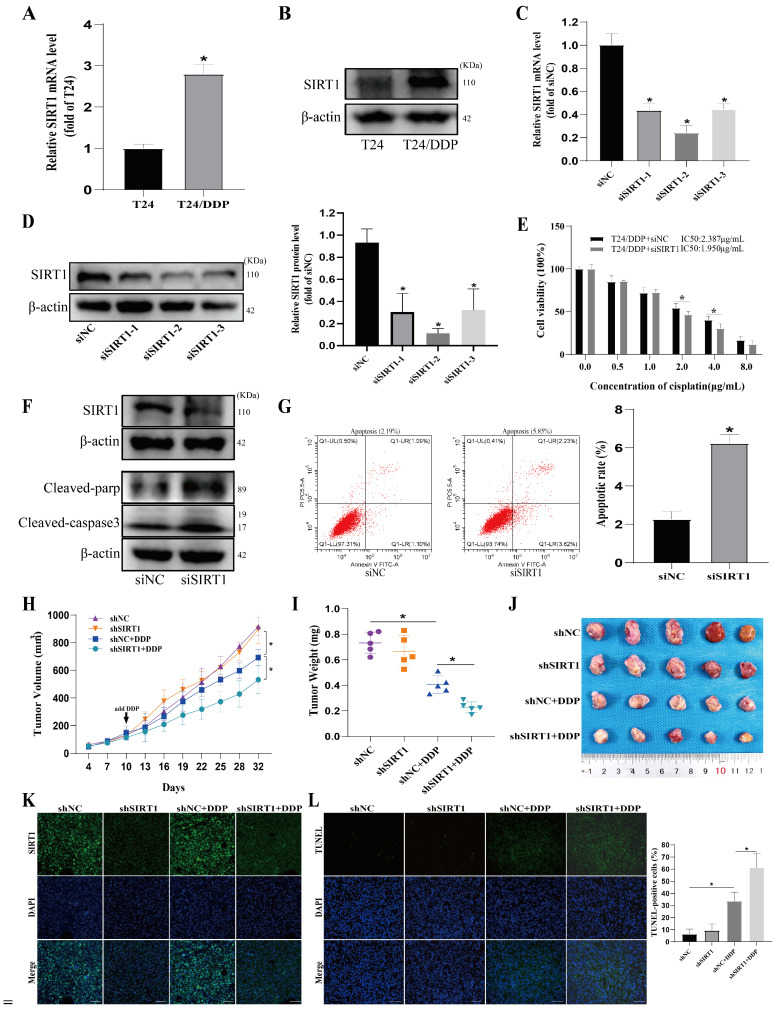
SIRT1 mediates cisplatin resistance in bladder cancer. (**A**) The mRNA expressions of SIRT1 in T24 and T24/DDP cell lines were determined with RT-qPCR. (**B**) The protein expressions of SIRT1 in T24 and T24/DDP cell lines were determined with Western blot. (**C**) The silencing efficiency of SIRT1 was assessed with RT-qPCR. (**D**) The silencing efficiency of SIRT1 was assessed with Western blot. (**E**) IC50 of the T24/DDP cell line against cisplatin was detected with CCK8 assay. (**F**) Expressions of SIRT1 and apoptosis marker proteins were determined with Western blot. (**G**) Apoptosis rate was determined with flow cytometry. (**H**–**J**) Tumor xenografts were established in BALB/c nude mice using T24/DDP cells stably transfected with SIRT1 shRNA or NC shRNA. Tumor size was recorded every three days. After one week, an equivalent amount of DDP (2.5 mg/kg) or 0.9% normal saline was intraperitoneally injected every 3 days. Tumor size (**H**), tumor weight (**I**), and tumor images (**J**) are shown (5 mice/group). (**K**) The expression of SIRT1 was detected with immunofluorescence; scale bar: 50 µm. (**L**) TUNEL staining to assess tumor cell apoptosis; scale bar: 100 µm. * *p* < 0.05 signifies a statistically significant difference.

It has been documented that SIRT1 can modulate autophagy through various pathways [17]. However, its role in regulating cisplatin resistance in bladder cancer through autophagy remains unclear. The RT-qPCR and Western blot results showed that both the mRNA and protein expression of SIRT1 in the T24/DDP cell lines were significantly higher than those in the T24 cell lines (Figure 3A,B). All three siRNA sequences of SIRT1 were able to silence SIRT1 expression significantly, with siSIRT1-2 having the most significant silencing effect on SIRT1 (Figure 3C,D). Therefore, in all the subsequent experiments, siSIRT1-2 was used to silence SIRT1. After SIRT1 silencing, the T24/DDP cells were found to be unable to maintain normal growth in medium containing 1 μg/mL of cisplatin; displayed significantly increased levels of both apoptosis (Figure 3G) and expressions of the apoptosis marker proteins Cleaved-parp and Cleaved-caspase3 (Figure 3F); and exhibited a decrease in the cisplatin resistance index (Figure 3E). To assess the impact of SIRT1-knockdown on tumor growth and DDP resistance in vivo, we introduced either control or SIRT1-knockdown T24/DDP cells into the axillary region of BALB/c nude mice, thereby establishing a xenograft model. After one week, an equivalent dose of DDP (2.5 mg/kg) or 0.9% normal saline was administered intraperitoneally at intervals of three days. Remarkably, in the DDP-treated mice, a noteworthy inhibition of the xenograft tumor expansion was displayed, manifesting as a concurrent reduction in both the tumor volume and weight, accompanied by an increase in the apoptosis rate of the tumor cells. Notably, the combination of SIRT1-knockdown with the DDP treatment elicited a more pronounced diminishment in both the tumor volume and weight, accompanied by a further increase in the apoptosis rate of the tumor cells (Figure 3H–L).

### 3.4. SIRT1 Mediates Cisplatin Resistance in Bladder Cancer via Autophagy Activation

**Figure 4:**
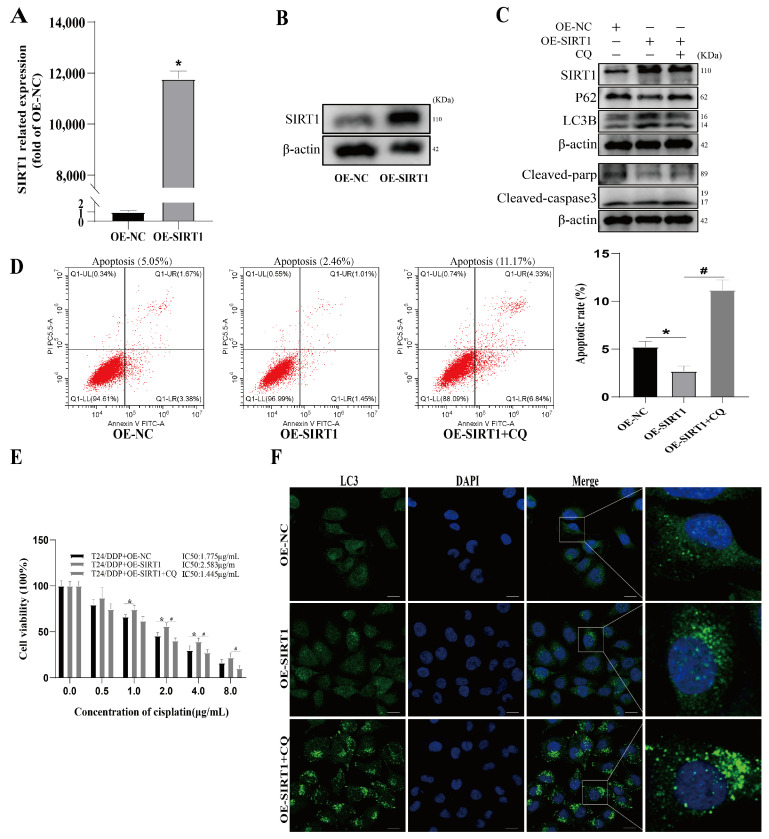
SIRT1 mediates cisplatin resistance in bladder cancer via autophagy activation. (**A**) The efficiency of SIRT1 over-expression was determined with RT-qPCR. (**B**) The efficiency of SIRT1 over-expression was determined with Western blot. (**C**) The expressions of SIRT1 and autophagy and apoptosis marker proteins were determined with Western blot. (**D**) Apoptosis rate was determined with flow cytometry. (**E**) IC50 of the T24/DDP cell line against cisplatin was detected with CCK8 assay. (**F**) Autophagosome formation was detected with immunofluorescence; scale bar: 20 µm. * *p* < 0.05 vs. OE-NC group, # *p* < 0.05 vs. OE-SIRT1 group.

To further explore the mechanism of SIRT1 in mediating cisplatin resistance, we overexpressed SIRT 1 through plasmid transfection. The RT-qPCR and Western blot results showed that both the level of the mRNA and protein expression of SIRT1 were significantly increased in the OE-SIRT1 group compared to in the control group (Figure 4A,B). SIRT1 over-expression significantly increased the level of autophagosome formation (Figure 4F), decreased P62 expression, increased LC3 expression (Figure 4C), led to a decrease in the apoptosis rate (Figure 4D), significantly decreased the expressions of Cleaved-parp and Cleaved-caspase3 (Figure 4C), and increased the cisplatin resistance index (Figure 4E). Blocking autophagic flux with 20 μmol/L of CQ partially reversed the cisplatin-resistance-promoting effect of SIRT1 over-expression.

### 3.5. SIRT1 Activates Autophagy via Beclin1 Deacetylation

**Figure 5:**
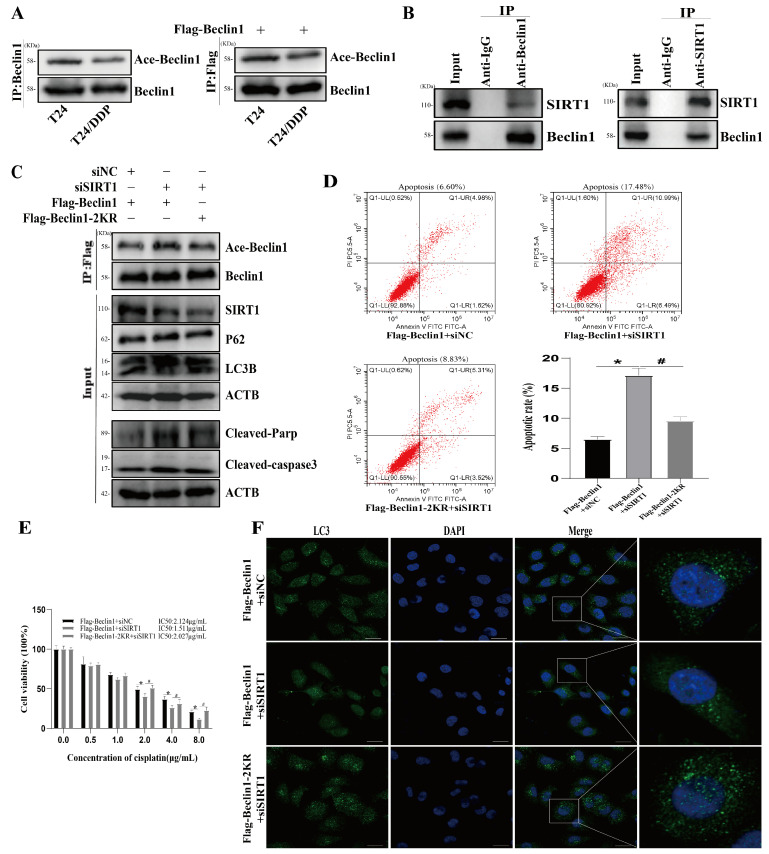
SIRT1 activates autophagy via Beclin1 deacetylation. (**A**) The acetylation of Beclin1 in the T24 and T24/DDP cell lines was determined with IP experiments. (**B**) The interaction of SIRT1 and Beclin1 was determined with CO-IP experiments. (**C**) The expressions of Beclin1 acetylation and autophagy and apoptosis marker proteins were determined with Western blot. (**D**) Apoptosis rate was determined with flow cytometry. (**E**) IC50 of the T24/DDP cell line against cisplatin was detected with CCK8 assay. (**F**) Autophagosome formation was detected with immunofluorescence; scale bar: 20 µm. * *p* < 0.05 vs. Flag-Beclin1 + siNC group, # *p* < 0.05 vs. Flag-Beclin1 + siSIRT1 group.

Beclin1 is a new substrate for SIRT1, thereby promoting the regulation of autophagy [6]. To determine the relationship between Beclin1 acetylation and cisplatin resistance, we investigated the acetylation level of Beclin1 in the T24 and T24/DDP cells with an IP assay, which showed that the Beclin-1 acetylation level was lower in the T24/DDP cells than that in the T24 cells (Figure 5A). To further determine the modifying effect of SIRT1 on Beclin1, we first determined the interaction between SIRT1 and Beclin1 using CO-IP experiments (Figure 5B), and then detected the acetylation of Beclin1 after silencing SIRT1. We found that SIRT1 silencing significantly increased Beclin1 acetylation (Figure 5B), reduced autophagosome formation (Figure 5F), increased P62 expression, decreased LC3 expression (Figure 5C), increased the apoptosis rate (Figure 5D), significantly increased the expressions of Cleaved-parp and Cleaved-caspase3 (Figure 5C), and decreased the cisplatin resistance index (Figure 5E). Recent research has revealed that Lys430 and Lys437 are acetylation sites in Beclin1, and the dual mutation of Beclin-1 (substitution of lysine 430 and 437 with arginine, 2KR) leads to the inhibition of its acetylation [6]. We maintained Beclin1 in a deacetylated state using transfected Flag-Beclin1-2KR, and found that silencing SIRT1, in this instance, did not increase the acetylation of Beclin1. The autophagy inhibition and reduced cisplatin resistance caused by SIRT1 silencing were partially reversed by the dual mutation of Beclin1.

## 4. Discussion

Bladder cancer is the most common malignancy in urology, and cisplatin-based chemotherapy is one of the primary treatments for this condition [18,19]. Cisplatin has been demonstrated to induce DNA damage, cell division, and apoptosis in cancer cells [20,21]. However, cisplatin resistance is a common issue in bladder cancer treatment, often leading to poor treatment efficacy [22]. Several studies have revealed that autophagy plays a crucial role in mediating cisplatin resistance [23,24,25], and inhibiting autophagy can enhance the sensitivity of bladder cancer cells to cisplatin [13]. In this study, we successfully developed cisplatin-resistant T24/DDP cell lines derived from bladder cancer cells. These cell lines were able to grow consistently in a culture medium containing 1 μg/mL of cisplatin. Autophagy plays a crucial role in maintaining cisplatin resistance in the T24/DDP cell line. The inhibition of autophagy has been shown to have significant effects on reducing the cisplatin resistance index of these cells and promoting cell apoptosis, aligning with the findings of Ji et al. [13]. Unfortunately, the clinical application of autophagy inhibitors is currently limited due to the importance of autophagy in various normal physiological processes. To overcome cisplatin resistance in bladder cancer, it is crucial to elucidate the regulatory mechanism of autophagy in resistant cells and explore potential targets for modulating resistance-associated autophagy. These investigations hold the key to improving the effectiveness of treatment.

Sirtuin 1 (SIRT1) is a member of the class III histone deacetylase, which is a nicotinamide adenine dinucleotide (NAD)-dependent histone deacetylase [26]. SIRT1 deacetylates histones and non-histone proteins involved in various cellular processes, including apoptosis, autophagy, DNA damage repair, and chemoresistance [27,28]. According to reports, the deacetylation of Foxk 2 by SIRT1 reduces tumor chemosensitivity to cisplatin [11]. SIRT1 can inhibit the chemosensitivity of HCC cells by activating autophagy [29]. However, the role of SIRT1 in promoting chemoresistance has rarely been reported in bladder cancer. We found that SIRT1 was highly expressed in the T24/DDP cell lines, and that SIRT1 silencing significantly reduced the cisplatin resistance index and increased the apoptosis rate in the T24/DDP cell lines. Consistent findings were replicated in our in vivo experiments, wherein the DDP treatment elicited a substantial inhibitory impact on the expansion of xenograft tumors. This effect was characterized as a concomitant decrease in both the tumor volume and weight, which was even greater with the combined approach of SIRT1 knockdown along with the DDP treatment. Interestingly, the effect of SIRT1 in promoting cisplatin resistance in the T24/DDP cells was positively correlated with the level of cellular autophagy. SIRT1 over-expression further increased the cisplatin resistance index of the T24/DDP cell lines and led to a significantly upregulated autophagy level. Blocking the autophagic flux with an autophagy inhibitor reversed the SIRT1-mediated increased resistance to T24/DDP and resulted in a significant increase in apoptosis. Therefore, we speculate that SIRT1 could promote cisplatin resistance in bladder cancer by activating autophagy, but the mechanism of its mediation of autophagy activation in T24/DDP cell lines remains elusive.

According to reports, SIRT1 plays essential roles in regulating the autophagy process through its deacetylase activity [30] and modifies substrates including various autophagy-related proteins, such as ATG 5, ATG 7, and Beclin1 [5,6,9]. Beclin1 is an important autophagy effector that has been found to mediate cisplatin resistance in bladder cancer by activating autophagy [13]. SIRT1 was reported to deacetylate Beclin1 at the lysine residues 430 and 437 in order to promote autophagosome maturation [6]. However, whether the acetylation of Beclin1 participates in the regulation of cisplatin resistance in bladder cancer remains unclear. We examined the acetylation of Beclin1 in the T24 and T24/DDP cell lines and found that the acetylation level of Beclin1 in the T24/DDP cell lines was consistently lower than that in the T24 cell lines, resulting in a negative correlation with cellular autophagy. To determine whether SIRT1 regulates autophagy in T24/DDP cells through deacetylating Beclin1, we first confirmed, using Co-IP experiments, that SIRT1 and Beclin1 could co-precipitate in the T24/DDP cells. Subsequently, we detected that the acetylation of Beclin1 was significantly increased after SIRT1 silencing, and both the autophagy and cisplatin resistance index were significantly reduced. However, the dual mutation of Beclin-1 (substitution of lysine 430 and 437 with arginine, 2KR) suppressed the increased Beclin1 acetylation and partially reversed the autophagy inhibition and decreased the cisplatin resistance index caused by SIRT1 silencing.

**Figure 6:**
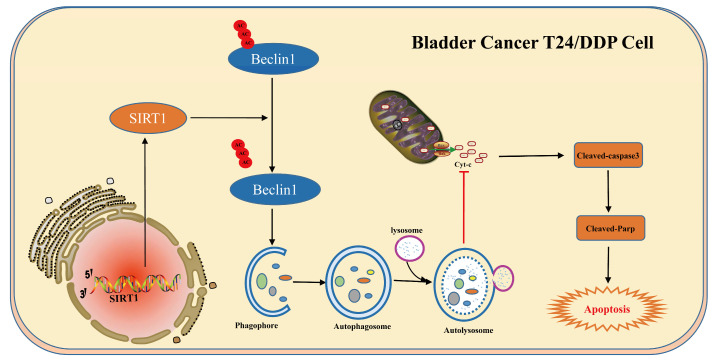
Diagram of cisplatin resistance mechanism in T24/DDP cell lines of bladder cancer. In the bladder cancer T24/DDP cell line, highly expressed SIRT1 promotes an increase in autophagic flux through Beclin1 deacetylation and subsequent inhibition of cisplatin-induced apoptosis.

In conclusion, we found that the deacetylase SIRT1 is highly expressed in T24/DDP cell lines, and can mediate protective autophagy activation through Beclin1 deacetylation, subsequently promoting cisplatin resistance in bladder cancer (Figure 6). This study provides new possible targets for overcoming cisplatin resistance in the treatment of bladder cancer.

## Figures and Tables

**Table 1:** Primer sequences used for RT–qPCR.

Gene Name	Forward Sequence	Reverse Sequence
SIRT1	5′-ACATAGACACGCTGGAACAGG-3′	5′-TCCTCGTACAGCTTCACAGTC-3′
ACTB	5′-CCTTCCTGGGCATGGAGTC-3′	5′-TGATCTTCATTGTGCTGGGTG-3′

## Data Availability

The data sets used and/or analyzed during the current study are available from the corresponding author on reasonable request.

## Supplementary Materials

The following supporting information can be downloaded at: https://www.mdpi.com/article/10.3390/cancers16010125/s1, Figure S1: The original western blot figures.

## Abbreviations

SIRT1: Sirtuin 1; NAD+: Nicotinamide Adenine Dinucleotide; BECN1: Beclin 1; IC50: half maximal inhibitory concentration; RI: resistance index; and siRNAs: small interfering RNAs.
