# Supplementary material for: SIRT1 Promotes Cisplatin Resistance in Bladder Cancer via Beclin1 Deacetylation-Mediated Autophagy
Source: Cancers (Basel). 2023 Dec 26;16(1):125. doi: 10.3390/cancers16010125 (PMC10778480; doi:10.3390/cancers16010125)

Figure S1. The original western blot figures.

Uncropped blots for Figure 2

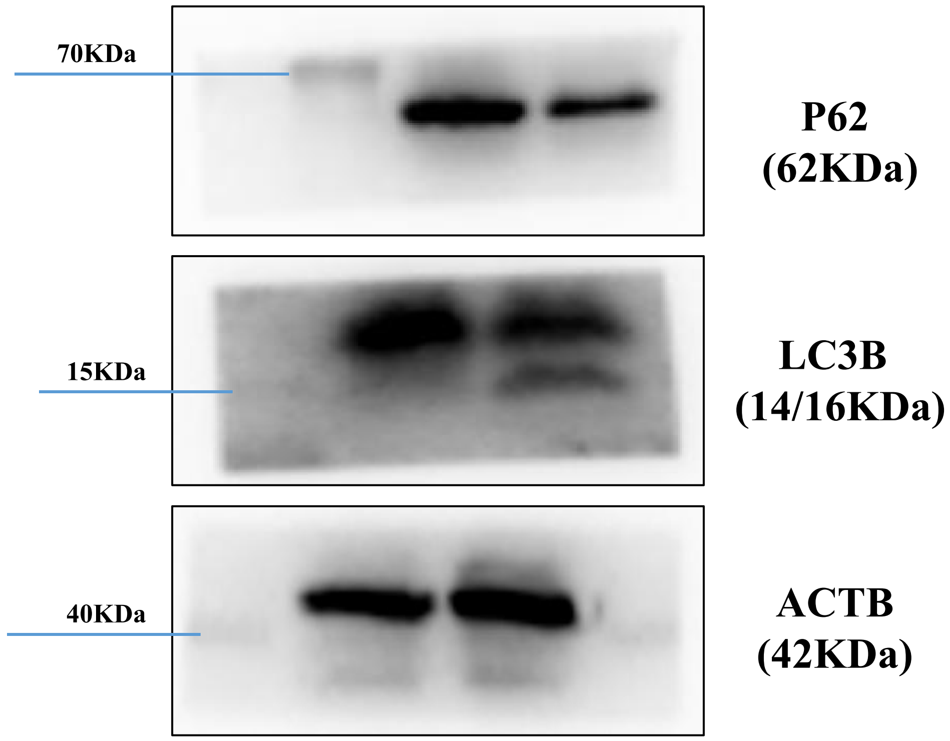

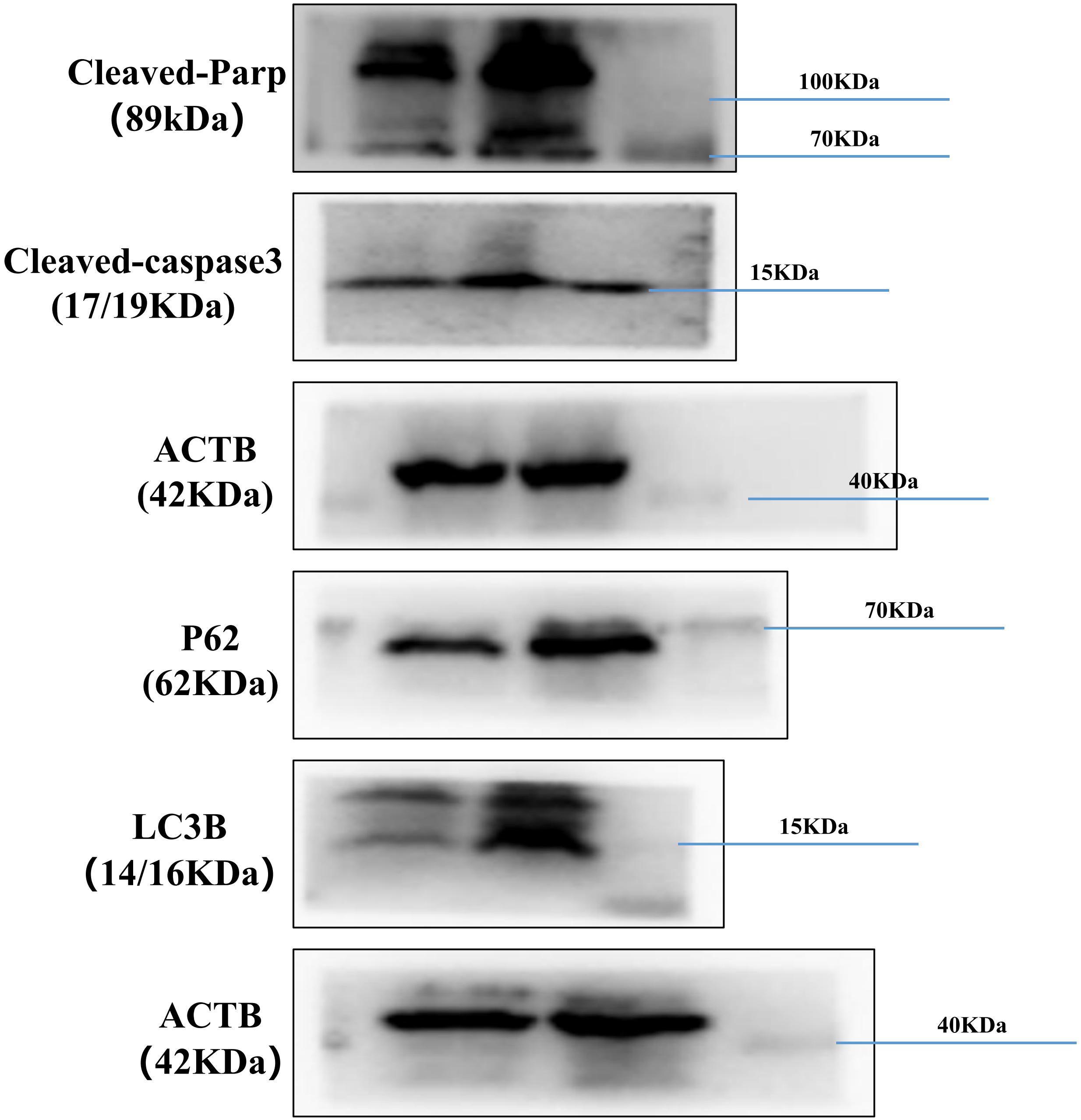

Uncropped blots for Figure 3

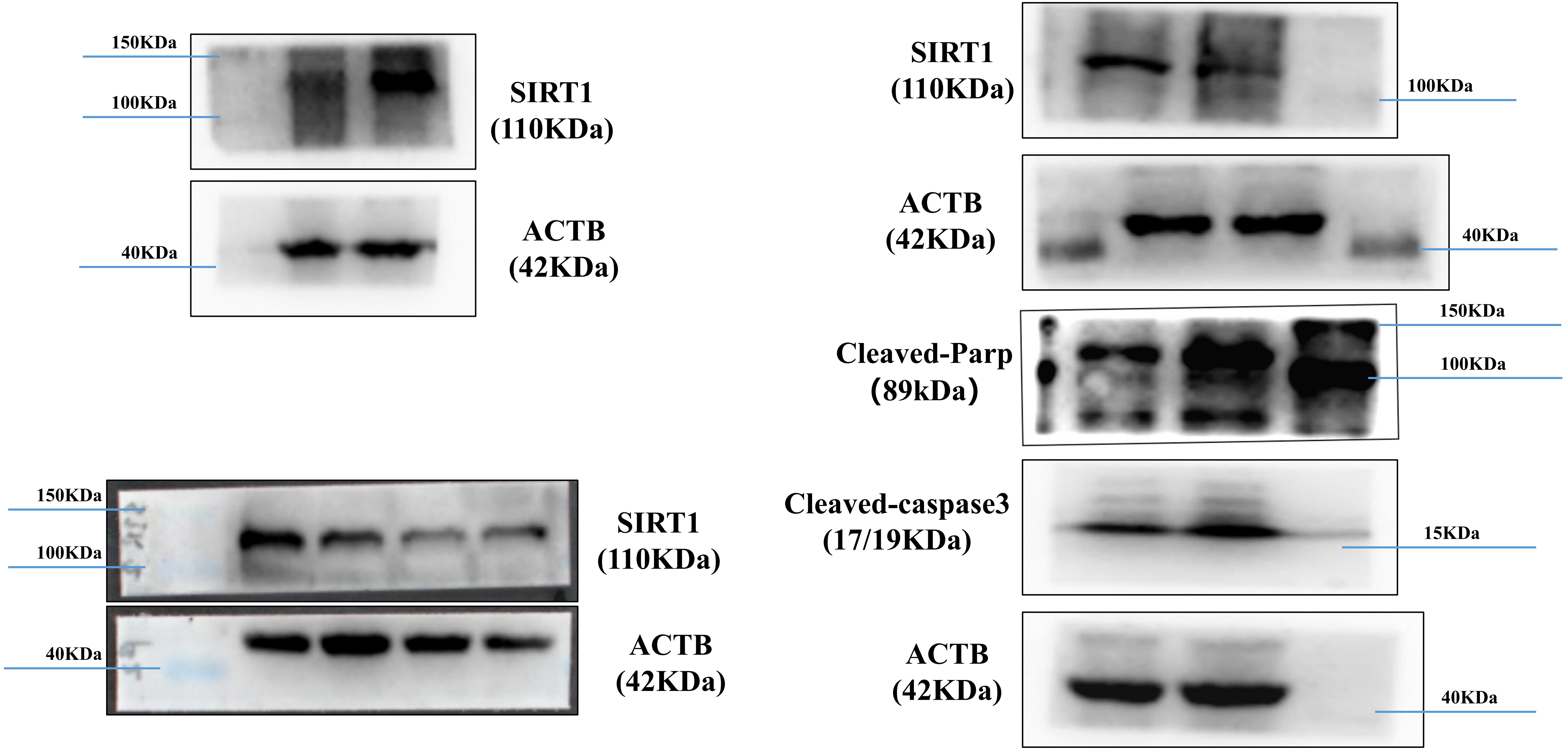

Uncropped blots for Figure 4

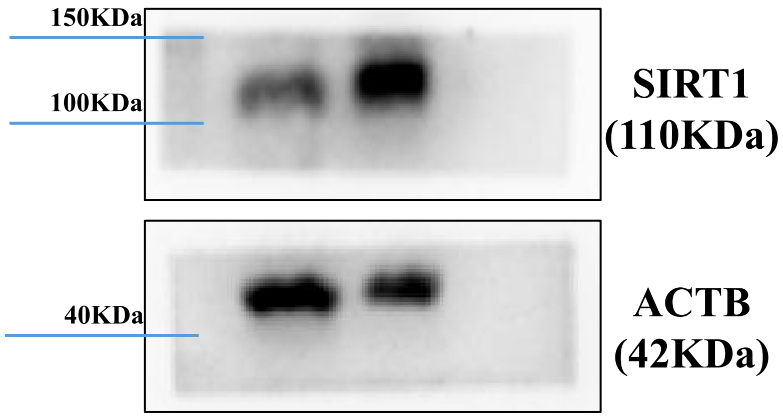

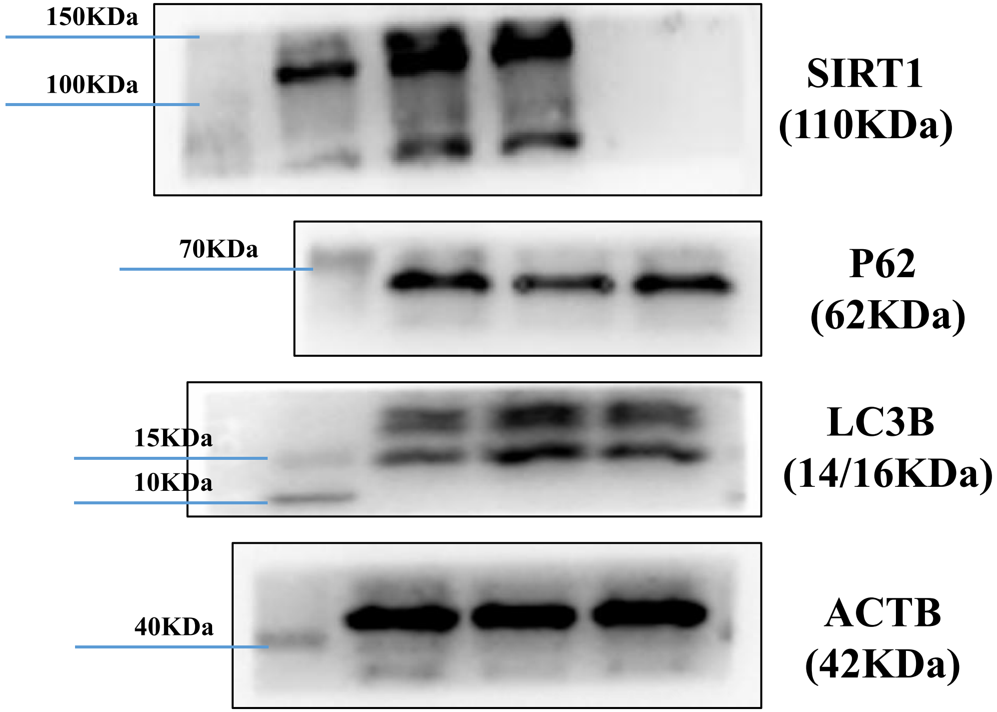

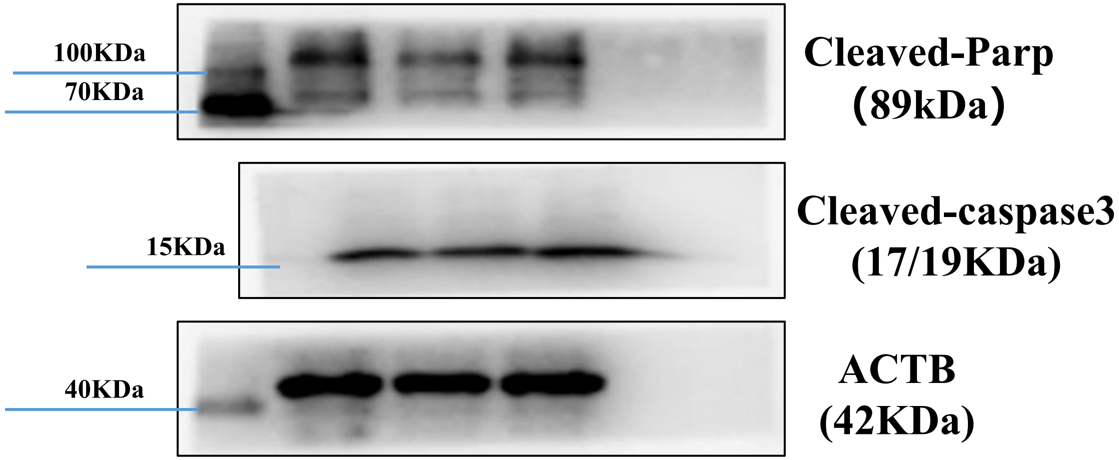

Uncropped blots for Figure 5

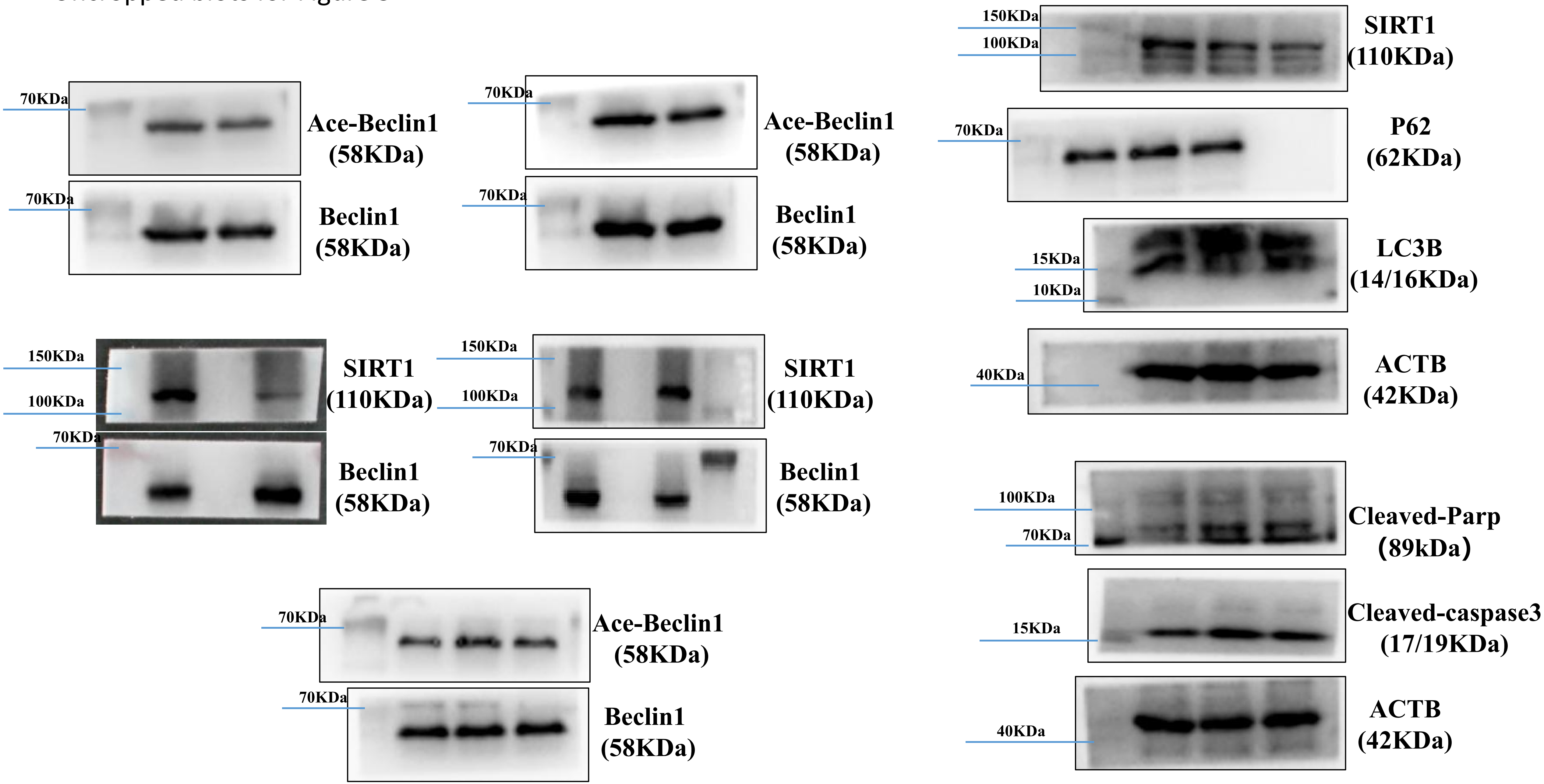

Supplement: Supplementary file 1 [file cancers-16-00125-s001.zip › cancers-2711929-supplementary.pdf]
